# Selected Papers from the 2017 International Conference on Micro/Nanomachines

**DOI:** 10.3390/mi9060284

**Published:** 2018-06-04

**Authors:** Fangzhi Mou, Jianguo Guan

**Affiliations:** State Key Laboratory of Advanced Technology for Materials Synthesis and Processing, International School of Materials Science and Engineering, Wuhan University of Technology, Wuhan 430070, China; moufz@whut.edu.cn

Thanks to their capabilities of converting various energy into motions, micro/nanomachines are believed to bring about revolutionary changes in many fields, such as biomedicine, micro/nanoengineering, and environmental monitoring and remediation. Even though remarkable progress has been made in the last few decades, there are still some great challenges, such as toxic fuels, low controllability, poor intelligence, single functions, etc., that need to be overcome so that micro/nanomachines can work safely and effectively in living bodies, natural water systems, and micro/nanofactories. In addition, swarmings and assemblies of micro/nanomachines which have collective behaviors may cooperatively perform complex biological or engineering tasks that cannot be completed by single ones. In this present Special Issue, we have published 10 papers, covering topics ranging from the design strategies, motion control, and applications of single micro/nanomachines [1,2,3,4,5,6,7], to the collective behaviors of micro/nanomachines [1,8,9,10].

Among the 10 published papers in this Special Issue, there are three reviews and four research papers that concern single micro/nanomachines. Ning et al. introduced the design from aspects of materials, geometries and fuels, the motion control strategies, and the potential applications of micromotors, and they outlined some future research directions [1]. Considering the unique advantages of tubular micro/nanomotors in driving force and surface functionalization, Zha et al. overviewed in detail the propulsion mechanisms, fabrication techniques, and applications, and pointed out some challenging problems of the existing tubular micro/nanomotors, as well as possible solutions to be explored in the near future [2]. Chen et al. summarized the recent advances in the design, manufacture, structural features, motion performance, and motion manipulation of light-powered micro/nanomotors and came up with some of their challenges and opportunities [3]. Jiao et al. reported a magnetic and fluorescent hybrid Janus micromotor by embedding magnetic nanoparticles and fluorescent dyes into the microparticles in a one-step process [4]. Sun et al. investigated the motion behaviors of self-powered liquid metal droplet machines under an external electric field, and put forward two non-dimensional parameters (Ä and Ö) to evaluate the ratio of the forces that resulted from the electric field to the fluidic viscous force, and the ratio of the friction force to the fluidic viscous force [5]. Li et al. proposed a self-propelled Janus foam motor, which can effectively integrate intriguing behaviors of the self-propulsion, efficient oil capture, and spontaneous self-assembly [6]. Feng et al. demonstrated three-dimensional (3D) control of the microrobot within a microfluidic chip using balanced magnetic and buoyancy forces, and the microrobot could grip particles (200 μm) and deliver it in a 3D space [7].

Motivated by the intriguing collective behaviors and the swarm intelligence of lives in nature, researchers are also dedicated to the understanding, construction, and manipulation of micro/nanomachine swarms and assemblies in this Special Issue. Shi et al. theoretically studied the pair dynamics of two self-propelled sphere dimers in the chemically active medium, which may shed light on the understanding of the collective dynamics of synthetic micro/nanomachines, as pair dynamics are the basic elements of the larger scale systems [8]. Ning et al. briefly introduced swarming, collective, and adaptive behaviors of interactive micromotors in the view of dynamic interactions between them [1]. Liu et al. summarized and compared the assembly and swarming of synthetic micro/nanomachines by the fuel induced methods (enzyme, hydrogen peroxide, hydrazine, etc.) and fuel-free induced approaches (electric, ultrasound, light, and magnetic) [9]. Zhang et al. streamlined the recent developments in light-controlled swarming and the assembly of colloidal particles based on the interactions that have arisen from optical forces, photochemical reactions, photothermal effects, and photoisomerizations, and also discussed the potential applications, challenges, and future prospects [10].

We wish to express our gratitude to all of the authors who submitted their papers to this Special Issue. We are also very grateful to all of the reviewers who helped us in an attentive and timely manner to improve the quality of this Special Issue.
